# Application of Deep Learning to Retinal-Image-Based Oculomics for Evaluation of Systemic Health: A Review

**DOI:** 10.3390/jcm12010152

**Published:** 2022-12-24

**Authors:** Jo-Hsuan Wu, Tin Yan Alvin Liu

**Affiliations:** 1 Shiley Eye Institute and Viterbi Family Department of Ophthalmology, University of California, San Diego, CA 92093, USA; 2 Wilmer Eye Institute, Johns Hopkins University, Baltimore, MD 21287, USA

**Keywords:** oculomics, artificial intelligence, machine learning, deep learning, retinal imaging, color fundus photograph, optical coherence tomography, systemic diseases, cardiovascular diseases, neurodegenerative diseases

## Abstract

The retina is a window to the human body. Oculomics is the study of the correlations between ophthalmic biomarkers and systemic health or disease states. Deep learning (DL) is currently the cutting-edge machine learning technique for medical image analysis, and in recent years, DL techniques have been applied to analyze retinal images in oculomics studies. In this review, we summarized oculomics studies that used DL models to analyze retinal images—most of the published studies to date involved color fundus photographs, while others focused on optical coherence tomography images. These studies showed that some systemic variables, such as age, sex and cardiovascular disease events, could be consistently robustly predicted, while other variables, such as thyroid function and blood cell count, could not be. DL-based oculomics has demonstrated fascinating, “super-human” predictive capabilities in certain contexts, but it remains to be seen how these models will be incorporated into clinical care and whether management decisions influenced by these models will lead to improved clinical outcomes.

## 1. Introduction

The retina is considered a window to the human body [1,2,3,4], as many systemic conditions have ocular manifestations, especially in the retina. The extensive correlations between retinal findings and systemic conditions can be attributed to the facts that the human retina is a direct extension of the central nervous system during embryonic development [5], and the retina is one of the most vascularized and metabolically active organs in the human body [6]. Characterization and quantification of retinal-systemic correlations is particularly valuable for gaining new insights, especially since the retina can be conveniently and readily imaged non-invasively using a variety of technologies. The term “oculomics” is coined to describe the clinical insights provided by correlating ophthalmic biomarkers with systemic health and diseases [1,7].

The most common retinal imaging modalities used in oculomics are color fundus photography and optical coherence tomography (OCT). Briefly, OCT performs high-resolution cross-sectional imaging of tissue structures in situ and in real time by measuring the time delay of light echoed from the tissue under examination [8,9]. The most common groups of diseases studied in oculomics are cardiovascular diseases (CVD) and neurodegenerative diseases (NDD) [1,10,11].

Oculomics studies concerning CVD typically involve color fundus photographs. For example, prior studies have shown that retinal vascular morphologies, such as vessel caliber and tortuosity, can help predict CVD risk factors [12], CVD mortality [13,14], and various major CVD events [15,16,17,18]. Similarly, retinal microvascular changes have been linked to higher risks of other systemic vascular diseases, such as kidney diseases and preeclampsia [19,20,21]. 

Oculomics studies concerning NDD typically involve OCTs. For example, retinal thickness measurements based on OCT have been used to diagnose and monitor multiple sclerosis (MS) [22,23,24]. Other studies have demonstrated an association between a thinner retinal nerve fiber layer (RNFL) and the diagnosis of Alzheimer’s disease (AD) [25,26,27,28,29], which accounts for more than 60% of clinical dementia. A major area of OCT-based oculomics is the early detection of pre-clinical NDDs.

Historically, retinal image annotation and feature labeling were performed either manually by humans or semi-automatically in oculomics. The process is time-consuming, labor-intensive and limited by intra/inter-reader imprecision. Recently, the advent of deep learning (DL) has revolutionized the field of oculomics. Briefly, DL, a subtype of machine learning (ML), is a representation learning method that uses multilayered neural networks (NN) to reiteratively adjust parameters and enhance performance [30,31,32,33]. DL is superior to classical ML techniques in image analysis, and has emerged as the leading ML technique for medical image classification. 

Medical subspecialities such as ophthalmology, with access to a large amount of imaging data, have been at the forefront of the DL revolution. Notably, DL has been shown to be on par with human experts in classifying various retinal diseases such as age-related macular degeneration and diabetic retinopathy [33,34,35,36,37,38,39], and the first FDA-approved fully autonomous system in any medical field is a DL-based system to detect diabetic retinopathy from color fundus photographs [40].

The retinal-systemic associations in oculomics were traditionally established using conventional statistical models or classical ML techniques. Given that oculomics primarily involves correlating ophthalmic biomarkers captured in retinal imaging with systemic conditions and that DL is the leading ML technique to analyze retinal images, the goal of this review is to summarize the latest literature in DL-based oculomics involving color fundus photography and OCT.

## 2. Literature Search Methods

The PubMed and Google Scholar databases were searched for published studies through July 2022, using individual and combinative search terms relevant to the this review. Major key words used included: (1) Deep learning-associated: “deep learning”, “machine learning”, and “neural network”; (2) Retinal imaging-associated: “ocular biomarkers”, “oculomics”, “ocular imaging”, “retinal imaging”, “fundus photographs”, “optical coherence tomography”; (3) Systemic disease/health-associated: “age”, “sex”, “demographic”, “systemic disease”, “systemic biomarkers”, “cardiovascular disease”, “neurodegenerative disease”, “stroke”, “multiple sclerosis”, “atherosclerosis”, “blood pressure”, “myocardial ischemia”, “dementia”, “Alzheimers disease”, “diabetes”, “renal disease” “kidney disease”, etc. 

No filter for publication year, language, or study type was applied. Reference of identified records were also checked. Studies applying DL on retinal-image-based oculomics to assess, predict, or diagnose systemic diseases and health biomarkers were considered relevant to the current review. Abstracts of non-English articles with relevant information were also included.

## 3. Results and Discussion

The following text is organized based on the imaging modality (fundus photography first, then OCT), and each sub-section is organized by the systemic parameter considered, with CVDs and their risk factors being the major focus.

### 3.1. Retinal Fundus Photography

Using retinal color fundus photographs from the UK Biobank and EyePACS, Poplin et al. published one of the first oculomics studies that demonstrated the ability of DL to predict systemic disease states and biomarkers [41]. In their study, a deep neural network (NN) showed reasonably robust performance in predicting major CVD events with an area-under-the curve (AUC) of the operating characteristic curve of 0.70. For reference, an AUC of 1.0 indicates perfect predictions, while an AUC of 0.5 indicates predictions no better than random chance. The deep NN was also capable of robust prediction of age (mean absolute error [MAE] ≤ 3.3 years), sex (AUC = 0.97), and smoking status (AUC = 0.71), etc. Regions of the color fundus photographs most activated during decision making by the deep NN were highlighted using attention maps [41]. For example, strong activation centered on the retinal blood vessels was seen during prediction for age and smoking status, while strong activation at the optic disc, retinal blood vessels and macula was seen during prediction for gender.

#### 3.1.1. Risk Assessment of CVD

Chang et al. presented a model that could generate a fundus atherosclerosis score (FAS) using DL-based retinal image analysis. The DL-generated FAS was then compared to the ground truth: a physician-graded score based on carotid ultrasonographic images. The DL model achieved an AUC of 0.71 in predicting the presence of carotid atherosclerosis [42]. Furthermore, by using the FAS to risk stratify patients, the authors found that cases in the top tertile (FAS > 0.66) had a significantly increased risk (hazard ratio = 8.33) of CVD mortality as compared to cases in the bottom tertile (FAS < 0.33). A similar CVD risk stratification study was performed by Son et al. [43]. They presented a model that could generate a coronary artery calcium score (CACS), by using DL-based retinal image analysis. The DL-generated CACS was compared to the cardiac computed tomography-derived CACS, and the model achieved an AUC > 0.82 in identifying cases with high CACS (CACS > 100). 

Khan et al., the DL model was trained to predict the presence of cardiac diseases from fundus photographs. With the electronic health record (EHR) as the ground truth, their model reached an AUC of 0.7 [44]. In another study, Cheung et al. used convolutional neural network (CNN) to segment the retinal vessels from fundus photographs and measured the vessel calibers [45]. They correlated the vessel calibers generated from DL-based segmentation with incident CVD events (defined as newly diagnosed clinical stroke, myocardial infarction or CVD mortality in EHR), and found that narrower calibers at certain vascular zones were associated with increased incident CVD risk. Lastly, a recent Chinese study trained a DL model to predict 10-year ischemic CVD risk using retinal image analysis [46]. Their estimation was compared with the calculation by a previously validated 10-year Chinese CVD risk prediction model, and an AUC of 0.86 and 0.88 was reported for predicting 10-year ischemic CVD risk ≥5% and ≥7.5%, respectively.

#### 3.1.2. Blood Pressure and Hypertension

In the study by Poplin et al., the DL model predicted diastolic BP (DBP) and systolic BP (SBP) with an MAE of 6.42 mmHg and 11.23 mmHg, respectively [41]. Subsequent studies published by different groups of authors showed similar results in that, in general, MAE of DBP (range: 6–9 mmHg) was smaller than that of SBP (range: 9–15 mmHg) [47,48]. Of note, a weak-to-moderate R^2^ ranged from 0.20 to 0.50 was observed for most DL models for BP prediction. Other studies attempted to train DL models to identify patients with hypertension [44,49,50]. The best result was reported by Zhang and colleagues using a cross-sectional Chinese dataset and neural network (NN) model [49]. Their model achieved an AUC of 0.77 in classifying patients with self-reported hypertension.

#### 3.1.3. Hyperglycemia and Dyslipidemia

The overall performance of DL models in estimating outcomes associated with hyperglycemia and dyslipidemia using retinal images was not robust. For the fundus-based prediction of HbA1c, the MAE reported in different studies ranged between 0.33–1.39%, with a low R^2^ of <0.10 in most studies [41,47,48]. Similar poor model performance and low R^2^ were observed for most DL models trained to predict blood glucose level and lipid profile [47,48]. An exception was a model developed by Zhang et al., which was able to discriminate patients with self-reported hyperglycemia and dyslipidemia from normal controls with an AUC of 0.88 and 0.70, respectively [49].

#### 3.1.4. Sex

Most DL studies predicting sex only performed internal validation, and in these studies, the models typically achieved an AUC of >0.95 during internal validation [41,47,48,51]. A notable exception was the study by Rim et al., in which the model was trained to predict multiple biomarkers, including sex. During external validation with 4 datasets obtained from patients of different ethnicities, this particular model predicting sex achieved an AUC ranging from 0.80 to 0.91 [47]. In the study by Korot et al., external validation was also performed using another local dataset, and their model achieved an accuracy of 78.6% [51].

#### 3.1.5. Age

For retinal-image-based prediction of age, most studies reported similar MAEs in internal validation, ranging from 2.43 to 3.55 years [41,47,48,52]. Khan et al. also trained the DL model to predict age > 70 years and reported an AUC of 0.90 for this task [44]. Interestingly, Zu et al. further calculated the retinal age gap, which was the difference between chronological age and the age predicted by DL [52]. Using mortality data in the national EHR, they found that each 1-year increase in the retinal age gap was associated with a 2% risk increase (hazard ratio [HR] = 1.02, *p* = 0.020) in all-cause mortality. This novel finding suggests DL-based retinal “age” may be a better marker for senescence on a tissue level than chronological age.

#### 3.1.6. Other Systemic Biomarkers and Disease Status

Other systemic biomarkers examined in DL-based oculomics included ethnicity, medication use, body composition, systemic organ functions, hematological parameters, and smoking status. Khan et al.’s model predicted ethnicity (Hispanic/Latino, non-Hispanic/Latino, others) based on fundus photographs using EHR as the ground truth, and reached an AUC of 0.93 [44]. Their model also showed a modest ability (AUC = 0.78–0.82) in identifying patients who take specific class of medications, such as angiotensin II receptor blockers and angiotensin-converting enzyme (ACE) inhibitors. In the study by Mitani et al., the DL model was trained to predict hemoglobin (Hb) and anemia, defined as Hb < 12 g/dL for women and <13 g/dL for men based on guidelines from the World Health Organization (WHO), using three types of data: retinal fundus images, participant metadata (race/ethnicity, age, sex and BP), and the combination of retinal images and metadata (multimodal data) [53]. The multimodal training data yielded the best model performance, with an AUC of 0.88 for anemia prediction and an MAE of 0.63 g/dL for Hb estimation. In contrast, the model trained only with retinal images yielded an AUC of 0.74 for anemia prediction and an MAE of 0.73 g/dL for Hb estimation. For the prediction of self-reported smoking status using fundus photographs, past studies [41,44,48,49,54] have reported models with AUC ranging from 0.70 to 0.86. As for the prediction of body mass index (BMI), most studies reported an MAE within 2–4 kg/m² and a low R^2^ < 0.30 [41,47,48].

Of note, Rim et al. reported an ambitious study that trained NN models to predict a total of 47 systemic biomarkers using retinal fundus photographs [47]. Although satisfactory results were achieved for sex (AUC = 0.96 in internal validation, AUC = 0.80–0.91 in external validation) and age (MAEs = 2.43 years in internal validation, MAEs = 3.4–4.5 years in external validation) prediction, the height prediction (MAEs = 5.5–7.1 cm), weight (MAEs = 8.3–11.8 kg), BMI (MAEs = 2.4–3.5 kg/m^2^), and creatinine (MAEs = 0.11–0.17 mg/dL) showed limited accuracy and generalizability in external validation with datasets of other ethnicities (R^2^ < 0.30 for all). Other biomarkers, such as C-reactive protein, thyroid functions, and blood cell counts, could not be predicted from retinal fundus images using DL in this study.

For chronic kidney disease (CKD) prediction, Sabanayagam et al. presented DL models that predicted the presence of CKD, defined as an estimated glomerular filtration rate (eGFR) < 60 mL/min per 1.73 m^2^, via retinal image analysis [55]. In their study, 3 model variations were trained: using only retinal fundus images, using only selected clinical data, and using both retinal images and clinical data (multimodal data). An AUC ranging from 0.73–0.84 and 0.81–0.86 was achieved for the retinal-image-only model and the multimodal data model, respectively, in external validation. Zhang et al. [56] presented a similar study that used 3 DL model variations to predict CKD. In external validation, an AUC ranging from 0.87–0.89 and 0.88–0.90 was reported for the retinal-image-only model and the multimodal data model, respectively. Additional analysis was performed to predict the eGFR values based on fundus photographs, and the DL models achieved an MAE ranging from 11–13 mL/min per 1.73 m^2^ (R^2^: 0.33–0.48) in external validation [56].

Tian et al. used retinal fundus images and DL techniques to predict the presence of Alzheimer’s Disease (AD) [57]. Patients diagnosed with AD were identified based on ICD codes in the EHR. The authors used DL techniques to segment retinal vessels, and then the segmentation maps were used for classification via a support vector machine (SVM). An overall accuracy of 82% (sensitivity: 0.79%, specificity: 0.85%) for discriminating normal subjects from subjects with AD was achieved. Saliency map analysis demonstrated that small retinal vessels were more prominently activated than large retinal vessels during decision making.

### 3.2. Optical Coherence Tomography

#### 3.2.1. Multiple Sclerosis (MS)

Compare to color fundus photographs, OCT is less commonly used in DL-based oculomics. Of the DL-based oculomics studies involving OCT, MS is the most studied systemic condition. In the study by Montolío et al., the performances of different ML algorithms, including linear regression, SVM, decision tree, k-nearest neighbors, Naïve Bayes, ensemble classifier and long short-term memory recurrent NN, in diagnosing MS and predicting the long-term disability course of MS were compared [58]. The diagnosis of MS was extracted from EHR and based on standard clinical and neuroimaging criteria (the McDonald criteria), [59] and the long-term disability ground truth was based on the expanded disability status scale (EDSS) scoring. All the ML models were trained with both clinical data and OCT-measured retinal nerve fiber layer (RNFL) thickness. The ensemble classifier, which performs prediction based on the weighted votes by various individual classifiers, [60] showed the best results for diagnosing MS (accuracy = 88%, AUC = 0.88), while the recurrent NN model showed the best prediction of long-term disability (accuracy = 82%, AUC = 0.82). In another study by López-Dorado et al., an NN model was also trained to diagnose MS using OCT images, with the ground truth determined by a neurologist based on the McDonald criteria [61]. Their model achieved a diagnostic accuracy of >90%. Additionally, they found the OCT-measured ganglion cell layer and whole retinal thicknesses to be the most discriminative features for diagnosing MS. 

#### 3.2.2. Age and Sex

Using OCT images centered on the optic nerve head and fovea, the MAE of DL-based age prediction ranged between 3.3–6 years, [62,63,64,65] with the best result reported by Hassan et al. [65]. Notably, in the study by Shigueoka et al., the CNN model revealed different correlations between the different retinal layers and age, [62] but this finding was not replicated in the study by Chueh et al. [64]. As for the OCT-based prediction of sex, accuracies and AUC ranged from 68% to 86% [63,64,65]. One study further compared the performances of DL models predicting sex using OCT foveal contour, OCT macular thickness, and infrared fundus photography, and showed the OCT foveal contour was most predictive [64].

Generally, as compared to color fundus photograph studies, OCT studies produced less robust DL models in predicting systemic biomarkers. Furthermore, most published OCT studies lacked external, independent validations. 

## 4. Conclusions and Future Direction

Most of the published studies to date only used a single imaging modality, e.g., either color fundus photograph or OCT, for model training. Ideally, multiple imaging modalities should be used simultaneously for model training. For example, in a recent study published in 2022 by Wisely et al., multimodal retinal imaging consisting of OCT, OCT angiography, and ultra-widefield pseudo-color and ultra-widefield autofluorescence images were used to train a CNN model in predicting symptomatic AD [66]. In addition to multimodal retinal imaging, tabular clinical data can also be incorporated into model training. For example, in the studies by Sabanayagam et al. and Zhang et al., incorporating relevant demographic data such as age, gender, ethnicity, etc. were found to improve the prediction of CKD from color fundus photographs [55,56]. However, the incorporation of multimodal retinal imaging and different data types into model training will inevitably increase the technical complexity from a machine learning point of view. “Detailed analysis of salient retinal regions/features associated with DL predictability will provide further insights into ocular-systemic relationships. Such information was only provided by a limited number of studies included in this review, most of which used DL to predict age, sex and CVD via color fundus images (Table 1). For future directions, it remains to be seen how these deep learning-based oculomics models will be incorporated into clinical care and whether management decisions influenced by these models will lead to improved clinical outcomes. 

## Figures and Tables

**Table 1:** Salient retinal fundus regions/features associated with deep learning predictions.

Study, Publication Year (Country)	Prediction Targets	Salient Regions/Features Identified
**Cardiovascular diseases (CVD) and CVD risk factors**		
Poplin et al. 2018 [41] (United States of America [USA])	5-year major adverse cardiovascular events	Retinal vessels (for major CVD risk factors)
Chang et al., 2020 [42] (Korea)	Carotid artery atherosclerosis	Optic disc and retinal vessels
Son et al., 2020 [43] (Korea)	Accumulation of coronary artery calcium	Central main retinal vessel branches
**Age**		
	Age	Retinal vessels
		Optic disc and retinal vessels
Zhu et al. 2022 [53] (China)		Peri-vascular regions
**Sex**		
Poplin et al. 2018 [41] (USA)	Sex	Optic disc and retinal vessels
Rim et al. 2020 [47] (Singapore)		Optic disc and retinal vessels
Korot et al. 2021 [51] (United Kingdom)		Fovea, optic nerve and vascular arcades

## Data Availability

Not applicable.
